# Cognitive requirements of cumulative culture: teaching is useful but not essential

**DOI:** 10.1038/srep16781

**Published:** 2015-11-26

**Authors:** Elena Zwirner, Alex Thornton

**Affiliations:** 1 Centre for Ecology and Conservation, University of Exeter, Penryn Campus, Penryn, TR10 9FE, UK; 2 Department of Genetics, Evolution and Environment, University College London, London WC1E 6BT, UK

## Abstract

The cumulative nature of human culture is unique in the animal kingdom. Progressive improvements in tools and technologies have facilitated humanity’s spread across the globe and shaped human evolution, but the cognitive mechanisms enabling cultural change remain unclear. Here we show that, contrary to theoretical predictions, cumulative improvements in tools are not dependent on specialised, high-fidelity social learning mechanisms. Participants were tasked with building a basket to carry as much rice as possible using a set of everyday materials and divided into treatment groups with differing opportunities to learn asocially, imitate, receive teaching or emulate by examining baskets made by previous chain members. Teaching chains produced more robust baskets, but neither teaching nor imitation were strictly necessary for cumulative improvements; emulation chains generated equivalent increases in efficacy despite exhibiting relatively low copying fidelity. People used social information strategically, choosing different materials to make their baskets if the previous basket in the chain performed poorly. Together, these results suggest that cumulative culture does not rest on high-fidelity social learning mechanisms alone. Instead, the roots of human cultural prowess may lie in the interplay of strategic social learning with other cognitive traits including the ability to reverse engineer artefacts through causal reasoning.

Human culture accumulates and increases in complexity over time, building on the achievements of previous generations in a ratchet-like manner^1^. This phenomenon, termed cumulative culture, generates ever-more efficient tools, technologies and social structures and has enabled human populations to colonize new niches and expand around the globe^2^,^3^. The cumulative nature of human culture stands in sharp contrast to the rest of the animal kingdom. While it is now clear that animal species across diverse taxa show simple forms of culture such as tools, foraging methods, vocal dialects and social rituals that spread through groups by social learning^4^,^5^,^6^, there is little compelling evidence that their cultural traits accumulate or improve over time. An understanding of the mechanisms that enable humans to accumulate cultural knowledge is thus central to our understanding of human evolution, ecology and psychology.

The dominant hypothesis advanced to explain the uniqueness of human cumulative culture posits that humans, unlike other species, rely on a set of social learning mechanisms that enable information to be transmitted with high fidelity such that cultural accomplishments can be built upon. In particular, prominent theorists point to the importance of imitation and teaching^1^,^7^,^8^,^9^,^10^. Imitation, the copying of precise body actions, is said to allow individuals to recreate the processes by which cultural artefacts are made, even if the process is novel and the task is causally opaque^1^. In contrast, other animals, including the great apes, are argued to rely predominantly on non-imitative social learning processes such as emulation, the copying of end-products^1^,^11^ (but see ref. 12 for a contrary view). According to this argument, apparent evidence for imitation in animals can generally be explained by attention to outcomes rather than processes and is limited to actions already within the observers’ behavioural repertoire, so cannot generate cumulative improvements. The second mechanism, teaching, involves knowledgeable individuals actively helping others to learn and its occurrence in humans is argued by some to rest on the ability to recognise and correct others’ ignorance^7^. This contrasts with all known examples of teaching in non-human animals, which are not thought to involve mental state attribution and are limited to a single adaptive context^13^, so cannot support the accumulation of new knowledge.

Although the proposal that imitation and teaching are necessary requirements for cumulative culture enjoys widespread acceptance, empirical evidence is scarce and equivocal^14^,^15^,^16^,^17^,^18^,^19^,^20^. In one recent study^15^, capuchin monkeys (*Cebus apella*), chimpanzees (*Pan troglodytes*) and human children were presented with a puzzle-box that could be solved in three successive stages to obtain increasingly valuable rewards. Only the children showed evidence of imitation and teaching and succeeded in reaching the last stage, providing support for the hypothesis that non-human primates lack the necessary cognitive requirements for cumulative culture. However, it is not clear that sequential problem-solving is equivalent to the cultural processes underlying technological improvements, or that the children would have been incapable of reaching the final stages had they been prevented from using teaching or imitation. Indeed, a recent touch-screen study on baboons using an iterated pattern-recollection task argued against the need for specialised human-specific cognitive mechanisms. Here, exposure to the sequence of choices made by others resulted in increases in performance and the emergence of distinct spatial patterns across chains of individuals^16^. In the context of material culture, a “transmission chain” experiment by Caldwell & Millen found that emulation was as effective as imitation and teaching in supporting cumulative culture^17^. Participants attempted to build paper aeroplanes that would fly as far as possible and were replaced one by one by new participants to simulate generational changes. The aeroplanes improved across all chains, regardless of whether participants were allowed to imitate, receive teaching or merely see the planes made by previous members of the chain.

Caldwell and Millen’s results suggest that teaching and imitation may not be necessary for cumulative culture in some contexts^17^, but the generality of their findings is open to question. Most people are likely to have made paper planes in the past, so results may be somewhat confounded by prior experience, and the task is relatively transparent, so may be particularly amenable to replication through emulation by copying fold marks on paper. Other studies provide mixed evidence that imitation or teaching are necessary in more unfamiliar and causally opaque tasks. Two recent studies point towards a role for imitation, but did not test the effects of teaching. In one, a transmission chain experiment involving the construction of a weight-bearing device using clay and reeds, imitation but not emulation resulted in cumulative improvements^18^. However here, in contrast to earlier work^17^, emulators had no information about the success of previous devices. Arguably, this may remove an important source of information available to emulators under natural conditions, where indications of tool efficacy may be gained through direct observation or associated cues such as fragments of food. In the other study, imitators but not emulators outperformed individual learners in making effective virtual fishing nets^19^. Nevertheless, all participants showed improvements across multiple trials, suggesting that imitation may not be strictly necessary for cumulative culture. Moreover, it is not known whether virtual tasks fully reflect the cognitive faculties involved in learning to manufacture real, physical artefacts (see refs 21,22). A more recent experiment^20^ called into question the importance of imitation, finding that in transmission chains starting with an expert flint-knapper, verbal teaching but not imitation or emulation improved naïve individuals’ performance. Owing to the specialised nature of the task, there was no improvement in performance across chains, although teaching slowed the degradation of the experts’ skills. Thus it remains rather unclear whether imitation and teaching are required for cumulative improvements in novel, real-world tool-making tasks.

The production of increasingly efficient tools is central to our species’ ecological success^3^,^21^,^22^. However, the cognitive requirements for cumulative cultural improvements in the manufacture of physical tools remain largely unexplored, with existing studies using computer-based tasks^16^,^19^,^23^, sequential puzzle-solving^15^ or the production of symbols or artefacts with no utilitarian function as tools^24^. The sole study of improvements in manufactured, functional tools to date^18^ did not examine the effects of teaching or allow social learners to appraise the efficacy of previous tools. We built on Caldwell & Millen’s experimental paradigm^17^ and presented chains of adult participants with the task of making a basket to carry as much rice as possible, using a set of 13 different materials. This variety of materials and their different possible uses were intended to create a more causally opaque end-result, while the task itself required participants to build a functional tool with which they were unlikely to have previous experience. While the task is relatively simple compared to, for example, flint-knapping or the manufacture of modern subsistence tools, it is intended to mimic conditions faced by our ancestors in the early stages of the emergence of cumulative culture.

To determine the efficacy of individual learning in generating improvements in tool efficacy we conducted an *Asocial* treatment in which participants had repeated opportunities to make baskets. We also manipulated opportunities for social learning in three experimental treatments. In the *Emulation* treatment participants could inspect previous baskets and were informed of the mass of rice these carried; in the *Imitation* treatment participants could observe the building process of others; and in the *Teaching* treatment participants who had already built their baskets could communicate verbally to help new group members. If emulation is insufficient to generate cumulative improvements in complex tools, as generally assumed, we predicted that only the Asocial, Imitation and Teaching chains would show evidence for improvement in basket efficacy. Moreover, based on theoretical predictions^9^,^25^ and results from previous tasks^18^,^19^, we expected Imitation and Teaching baskets to outperform those of asocial learners.

## Methods

### Participants

In total, 190 participants took part. Of these, 180 were randomly assigned to one of three social learning treatments: Emulation, Imitation and Teaching. Within each of these three treatments we divided participants into 10 replicate transmission chains, with each chain consisting of six participants. The remaining 10 participants were assigned to the Asocial treatment, where they made 6 consecutive baskets with no opportunity to learn from others. Participants’ ages ranged from 17 to 69 (mean ± SD = 29.2 ± 0.8; one participant did not provide age); 104/190 were female. Participants were recruited at the University of Exeter’s Penryn Campus, Penryn College, Truro College and University College London. To incentivise participation and performance in the task, a prize of £60 was offered to the three best performing groups and an extra prize of £20 was offered to 3 participants with a raffle.

### Ethical statement

This study was carried out in accordance with the ethical standards of the 1964 Declaration of Helsinki and the guidelines of the British Psychological Society’s Code of Human Research Ethics. All methods were approved by the University of Exeter Biosciences Research Ethics Committee and all participants provided written, informed consent before taking part in experiments.

### Procedure

Experiments were conducted in lecture theatres and classrooms, with screens separating areas for building and testing of baskets. In the building area, two desks facing opposite directions were prepared with a pair of scissors and the materials for the building task (Table 1). The testing area comprised a “filling area” with bowls full of dry rice (totalling 7 kg) and a scoop, with a large empty bowl positioned on a digital scale (±1 g precision) 5 m away.

Before starting the experiment, participants were informed that they were about to take part in a manual construction task and asked to complete a consent form. Participants in transmission chain treatments were randomly assigned a number from one to six indicating their position in the chain. This introduction phase took place in the testing room for each individual in the Emulation and Asocial treatments. In the Imitation and Teaching treatments participants were gathered in their groups of six and told that they would be asked to enter the testing room one by one.

### Building phase

Once in the test room, participants were given written instructions: to build, within a maximum of five minutes, a basket to carry as much rice as possible using any of materials provided. For participants in the Asocial treatment, the instructions explained that after building and testing their first basket they would then be required to build and test a further five baskets (i.e. build and test a total of six baskets). A stopwatch showing the time elapsed was displayed in view of the participants, who were also verbally updated of the build-time remaining as each minute elapsed.

### Testing phase

Once the building time had expired, the basket was tested behind a screened area, out of view of other participants. Participants were asked to load as much rice as they dared and carry it to the weighing bowl on the scale positioned at 5 m distance. This was done twice to test the resistance of the basket. The mass of rice carried was recorded for each trial. We also noted whether the basket broke during transportation or while pouring rice into the bowl; if the basket collapsed during transportation, we recorded the distance rice was transported. After their basket had been tested, participants were asked to complete a post-study questionnaire and debrief form and told that they could leave immediately (Asocial, Emulation and Imitation treatments) or asked to remain behind to help other group members (Teaching treatments). No time restriction was given for the testing part of the experiment.

### Experimental treatments

**Figure 1:**
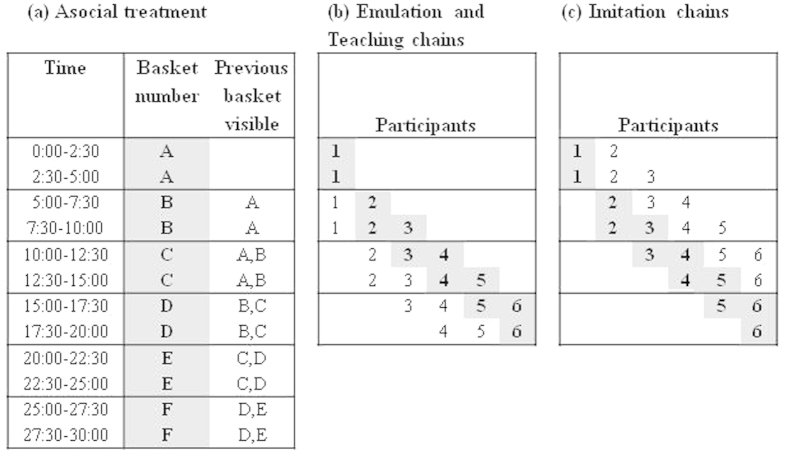
(**a**) Experimental design for Asocial treatment. Each participant built six baskets in succession (A–F; rounds of building indicated by grey cells; time given in minutes). At each round of building, up to two of the participant’s previous baskets were left on display. (**b,c**) Experimental design in the three transmission chain treatments. Participants (from 1 to 6) are engaged in different roles (identified by shadings) in particular time windows. Grey cells represent participants engaged in building. White cells represent (**b**) participants’ baskets on display in Emulation chains or previous participants teaching builders in Teaching chains; (**c**) participants observing builders in Imitation chains.

Before starting the experiment, all participants were given written and spoken instructions relevant to their allocated treatment group. The experimental procedures for the four treatments are shown in Fig. 1. Participants in Asocial treatments were instructed to build and test six baskets in succession without communicating with or observing others building or testing baskets. Up to two of the participant’s previous baskets were left on display at each new round of basket building (Fig. 1a).

Participants in the Emulation (Fig. 1b) treatment were informed that they were not allowed to communicate with or observe others building baskets, but (with the exception of the first chain member) would be allowed to observe and inspect completed baskets from previous participants in their chain. When presented with completed baskets, participants were told which member of the chain made it, the amount of rice it carried and the meters covered.

In the Imitation treatment (Fig. 1c), participants were informed that they could not communicate with others about the task, but (with the exception of the first chain member) would be present and could observe while earlier chain members built their baskets. After five minutes observation, they were shown their materials and asked to start building. Once the building time had passed, the basket was tested behind a screened area as above. Participants were then asked to complete a post-study questionnaire and told that they could leave. Participants were not informed of the efficacy of baskets.

In the Teaching treatment (Fig. 1b), participants were instructed to return to the building area to help other group members after testing their own baskets. They were informed that they could communicate with others, but were not allowed to physically help build the basket for others or touch any of the materials. After the five minutes in the teaching role, participants were asked to leave the test area. Participants engaged in building (except for the first participant) were informed that they could communicate with teachers but were not allowed to see others building baskets.

### Statistical analyses

All statistical analyses were conducted in Genstat v.16. Multifactorial analyses were conducted using Linear Mixed Models (LMM) or Generalised Linear Mixed Models (GLMM) for normal or non-normal data respectively. We confirmed the assumptions of homogeneity of variance and normality of error through inspection of model residuals. Each set of six baskets made by individual participants in Asocial chains and each chain of six baskets in transmission chain treatments was assigned a unique Group number, which was fitted to models as a random term. For convenience, we refer to the positions of baskets within each group (1–6) as “generations”. We tested whether baskets’ efficacy improved across the chains using a LMM with the total mass of rice carried by each basket fitted as the response term. Generation, experimental treatment (Asocial, Emulation, Imitation or Teaching) and the number of materials incorporated into the basket were fitted as explanatory terms. To analyse the effects of treatment on basket robustness, we used GLM with a binomial error structure indicating the proportion of baskets that broke in each group. Excluding the first basket in each group of six, the total number of baskets (5) was fitted as the denominator, with the number of baskets that broke as the numerator.

To compare the complexity of baskets across treatments, we analysed the total number of materials used to make each basket using a GLMM with Poisson error structure. Finally, we used a GLMM with Poisson error structure to examine the factors influencing fidelity in the materials used to make baskets across chains. As the response term, we fitted the number of changes in the materials used for each basket compared to the previous basket in the chain (i.e. the total number of new materials used plus previously used materials excluded; data do not include the first basket in each chain). Treatment was fitted as an explanatory term, to test the prediction that emulation results in lower fidelity copying than imitation and teaching. In Asocial, Emulation and Teaching chains basket-builders may act on knowledge of the efficacy of previous baskets (using their own experience or information provided by experimenters or previous chain members respectively). To test whether participants in these three treatments made strategic building choices, using new materials when the previous basket performed poorly, we also fitted the total mass of rice carried by the previous basket as an additional explanatory term.

## Results

### Mass of rice carried

**Figure 2:**
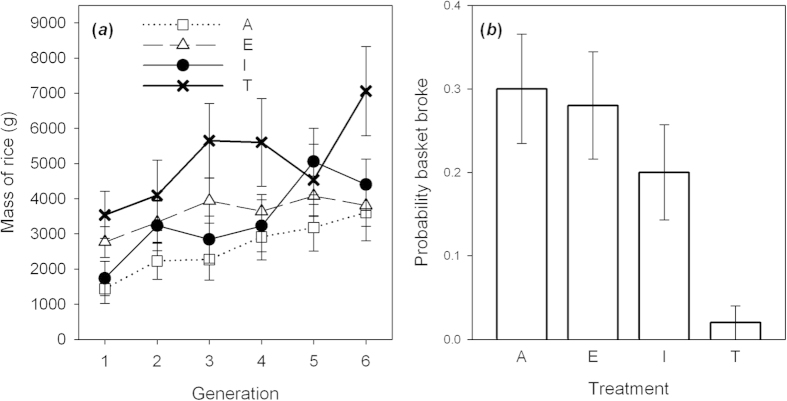
(**a**) Improvements in basket efficacy across chains in the four treatments (Asocial: open squares; Emulation: open triangles; Imitation: solid circles; Teaching: crosses). Points are means ± S.E. from raw data. (**b**) Probability of basket breakage across the four treatments (A: Asocial; E: Emulation; I: Imitation; T: Teaching). Bars show means ± S.E.

All four treatments showed similar improvements along chains. LMM analysis showed that the mass of rice increased across generations (*F*_1, 199_ = 49.24, *p* < 0.001; Fig. 2a; response variable normalized for analyses with a square root transformation), but there were no significant differences between treatments (*F*_2, 36_ = 2.51, *p* = 0.074; treatment*generation: *F*_2, 196_ = 2.03, *p* = 0.112). The number of materials used to build baskets had no effect on their efficacy (*F*_1, 217_ = 0.00, *p* = 0.992).

The results for every group are shown in Supplementary Figure S1 online. The last basket was more effective than the first for all ten participants in Asocial treatment (paired t-test comparing rice carried by first and last basket: *t* = 4.46; *p* = 0.002), in eight of the ten Emulation chains (*t* = 2.58; *p* = 0.03), eight of the Imitation chains (*t* = 3.46; *p* = 0.007), and seven of the Teaching chains (*t* = 2.94; *p* = 0.017). There were no differences between treatments in the difference between the mass of rice transported between the first and the last basket in each group (ANOVA: *F*_2, 27_ = 1.66; *p* = 0.170). However, although we detected no overall differences between treatments, it is notable that Teaching chains produced a disproportionate number of the best-performing final generation baskets, occupying six out of the ten top ranks (binomial test, expected proportion = 0.25; *p* = 0.028; Supplementary Table S1).

### Durability

Excluding the first participants, who had no opportunities for trial and error or social learning, the proportion of baskets within each group that broke during testing was significantly different between treatments (GLM: *χ*^2^_3_ = 9.38; *p* = 0.025; Fig. 2b). This difference was driven by greater durability of baskets in Teaching chains than in the other three treatments. Across all Teaching chains, there was only a single instance of basket breakage. In contrast, there were basket breakages for six of the 10 Asocial participants (15/50 broken baskets in total; two-sample binomial test comparison to Teaching chains, *p* < 0.001), eight of the Emulation chains (14/50 baskets; binomial comparison to Teaching chains, *p* < 0.001), and five of the Imitation chains (10/50 baskets; binomial test comparison to Teaching chains, *p* = 0.004). Excluding Teaching chains, there were no significant differences in the proportion of broken baskets in the other treatments (GLM: *χ*^2^_2_ = 1.44; *p* = 0.486).

### Use of materials

**Figure 3:**
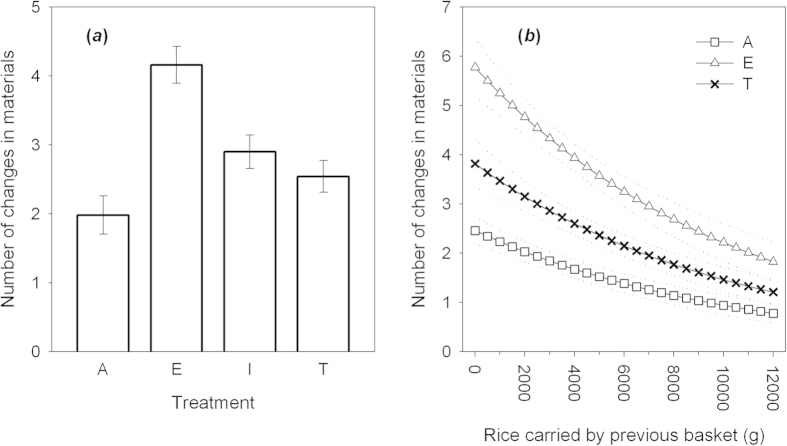
(**a**) Mean number of changes in materials ( ± S.E.) at each step in the chain across the four treatments (**b**) Number of changes in materials as a function of the mass of rice carried by the previous basket in Asocial (squares) Emulation (triangles) and Teaching groups (crosses). Lines are predicted means ± S.E. from GLMM analysis.

The number of materials used to build each basket did not change significantly within groups (effect of generation: GLMM: *χ*^2^_1_ = 0.23, *p* = 0.632) and did not differ between treatments (*χ*^2^_3_ = 3.44, *p* = 0.343). However, treatments differed significantly in the variability of materials used within chains, with baskets in emulation chains showing more changes in material usage from one step in the chain to the next compared to other treatments (GLMM, effect of Treatment: *χ*^2^_3_ = 17.94, *p* = 0.002; Fig. 3a; excluding Emulation chains the other treatments did not differ: *χ*^2^_2_ = 3.18, *p* = 0.224). Moreover, in Asocial, Emulation and Teaching chains (where participants had information about previous baskets’ efficacy), participants made a greater number of changes in materials when the previous basket performed poorly (GLMM treatment*rice carried by previous basket: *χ*^2^_2_ = 13.18, *p* = 0.001; Fig. 3b).

## Discussion

Our experiment demonstrates an important advantage for teaching in cultural transmission but adds to the growing body of evidence suggesting that specialised social learning mechanisms alone are insufficient to explain the emergence of cumulative technology in the human lineage^16^,^17^,^22^,^26^. Within only six experimental “generations”, baskets became increasingly effective at carrying large quantities of rice, regardless of participants’ opportunities to engage in trial and error learning or learn socially through imitation, emulation or teaching. It is particularly notable that emulation of end-products was sufficient to drive cumulative improvements in functional, multi-component tools, mirroring findings from the simpler, more familiar task of paper aeroplane construction^17^. Theorists have long argued that cumulative culture depends on high-fidelity social learning mechanisms to prevent information from degrading at each stage of the transmission process (e.g. refs 1,7,9,25). Our results confirm that emulation results in lower-fidelity copying than imitation or teaching^1^,^7^: compared to other chains, participants in emulation chains made significantly more changes in the materials used to make baskets from one step in the chain to the next. However, contrary to theoretical predictions, this low fidelity did not prevent the emergence of cumulative improvements equivalent to those in higher-fidelity treatments. Thus, at least in some contexts, high-fidelity copying may not be a necessary requirement for cumulative culture.

Nevertheless, it is clear from our experiment that teaching can provide important cultural benefits. Baskets from teaching chains appeared to be over-represented among the top performers in the final-generation, raising the possibility that teaching may have outperformed other treatments had our transmission chains been longer. More strikingly, we found that baskets were significantly less likely to break if the manufacturer had received teaching. Under natural conditions, such increased durability would provide important advantages, both in enabling long-term re-use of tools and in ensuring that they remain available as learning models for future generations. While many authors have speculated about the importance of teaching in human material culture (e.g. refs 7, 8, 9, 10,27), direct experimental evidence has been limited to Morgan *et al.*’s finding that teaching promotes skill acquisition in a flint-knapping task^20^. Our results concur broadly with this finding, raising the prospect that verbal teaching may have been advantageous in hominin tool construction even before the invention of stone tools. In our experiment teaching may have been particularly effective in promoting tool robustness compared to other forms of social learning because teachers could communicate about aspects of design that are not apparent through observation alone and may only be revealed through experience of using the tool. We speculate that teaching groups produced more robust baskets than asocial learners because participants benefitted from the collective knowledge of their predecessors^28^ and were more cautious given that they only had a single attempt at basket-making.

According to some definitions, cumulative culture entails behaviour that no individual could invent in their lifetime^7^,^9^. From this perspective, one might argue that our experiment does not demonstrate cumulative culture, given that asocial learners’ baskets were as effective as those in other experimental treatments. However, this definition is problematic for a number of reasons. From a practical point of view, a requirement for behaviour beyond an individual’s capabilities effectively eliminates the potential for experimental research as participants would be incapable of completing the task. From a conceptual point of view, it obscures the factors at play in the early emergence of cumulative culture in the hominin lineage, when technological achievements were still within the capabilities of individuals. Indeed, our results suggest that cultural processes cannot be divorced from individual cognitive traits.

The success of participants in creating and improving baskets points to the importance of reasoning about causal relationships in generating effective, goal-directed outcomes. The ability to represent and predict causal outcomes is likely to have allowed naïve individuals to plan and construct baskets for the first time and, for asocial learners, contributed to the refinement of designs over subsequent attempts. Indeed, experimental studies of lithic technology highlight the importance of individual practice in predicting and controlling the consequences of motor actions^29^. Causal reasoning is also likely to be central to the efficacy of emulation. An individual encountering a discarded tool alongside evidence of its purpose (e.g. remnants of extracted food) may reverse-engineer the construction by making inferences about both the relationship between the tool’s physical form and its function, as well as the intentions of the person that created it^22^,^30^. Such reverse engineering becomes increasingly implausible for artefacts whose construction is causally opaque^10^,^20^, but the earliest human tools are thought to have been relatively simple constructions made from easily workable, perishable materials^31^ and are likely to have been amenable to reconstruction via emulation. Understanding the processes involved at these early stages in human cultural evolution is a critical step in explaining later diversification and improvements in technology. Our relatively simple task is unlikely to fully capture the cognitive requirements for building the sophisticated tools found in the archaeological record or in modern subsistence societies, but instead may reflect the processes at play at the dawn of human cumulative culture, when our ancestors first began to build and improve on simple, multi-component tools. A growing reliance on increasingly complex tools may subsequently have favoured selection for more sophisticated cognitive processes, which in turn enabled yet further cultural achievements and so on, up to a point where the products of culture became too complex to reverse-engineer through emulation alone.

Our results also support theoretical predictions that complex culture requires strategic assessments about the potential advantages of using social information^32^,^33^. Cumulative cultural change rests not only on social learning, but also on the innovative incorporation of changes to existing designs^34^. In our experiment, participants were increasingly likely to make changes to the materials used to make baskets if the previous basket was relatively ineffective. This ability to take into account the success of others’ efforts when deciding whether to copy is not unique to humans (e.g. see ref. 35 for an example in birds), but in combination with causal reasoning processes it may be instrumental in permitting the accumulation of technological improvements even in the absence of opportunities to imitate or receive teaching.

To conclude, our results cast doubt on the widely accepted hypothesis that imitation and teaching are fundamental prerequisites for cumulative culture^1^,^7^,^8^,^9^,^10^. The active involvement of a knowledgeable teacher can clearly generate important advantages, but it is not a limiting factor. We find that emulation is sufficient to generate cumulative improvements, but argue that it cannot be considered in isolation from other cognitive factors such as causal reasoning and strategic decision-making. If we are to understand the causes of the cultural chasm between humans and other species, we must extend our focus beyond social learning mechanisms and conduct a broad examination of the minimal cognitive requirements for cumulative culture.

## Additional Information

**Accession codes:** Raw data files have been deposited in Figshare: doi: 10.6084/m9.figshare.1363818.

**How to cite this article**: Zwirner, E. and Thornton, A. Cognitive requirements of cumulative culture: teaching is useful but not essential. *Sci. Rep.* **5**, 16781; doi: 10.1038/srep16781 (2015).

## Supplementary Material

Supplementary Information

## Figures and Tables

**Table 1:** List of materials given to each participant.

Quantity	Material and dimensions
2	String (40 cm)
1	Fabric gauze (25 × 27 cm)
1	Sheet of newspaper
1	Bubble wrap (40 × 10 cm)
1	Wooden stick (42 × 1.5 × 1.5 cm)
2	Bottle tops
2	Strips of adhesive tape (42 cm)
3	Drawing pins
3	Rubber bands
2	Drinking straws (21 cm)
2	Skewers (25 cm)
1	Paper napkin
1	Stapler with staples
