# Supplementary material for: Cognitive requirements of cumulative culture: teaching is useful but not essential
Source: Sci Rep. 2015 Nov 26;5:16781. doi: 10.1038/srep16781 (PMC4660383; doi:10.1038/srep16781)

SUPPLEMENTARY MATERIAL

Cognitive requirements of cumulative culture: teaching is useful but not essential.

Elena Zwirner[1,2] & Alex Thornton[1]

[1]Centre for Ecology and Conservation, University of Exeter, Penryn Campus, Penryn, UK

[2] Department of Genetics, Evolution and Environment, University College London, UK

Table S1: Top ten most successful final-generation baskets

Rank	**Mass of rice carried (g)**	**Treatment**
1	12070	T
2	12015	T
3	11008	T
4	10228	T
5	9393	A
6	8916	I
7	7548	E
8	6445	T
9	6360	T
10	5827	I

Figure S1: Basket efficacy across all 10 experimental groups in every treatment. A: Asocial; E: Emulation; I: Imitation; T: Teaching. Separate LMM analyses on each treatment confirmed that across all treatments the mass of rice carried increased across generations; statistical results are given in the legends. Group number was fitted as a random term.

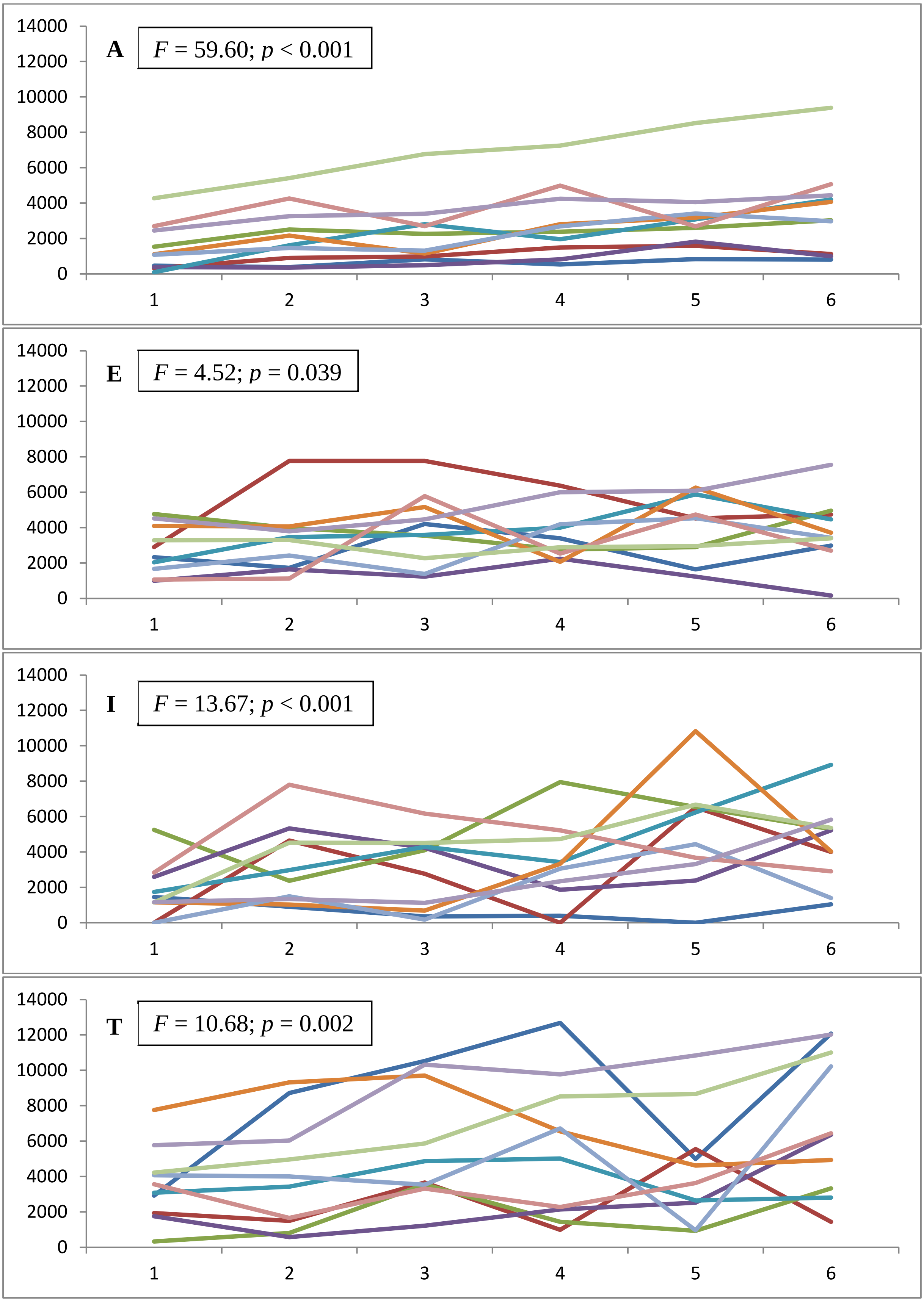

Supplement: Supplementary Information [file srep16781-s1.pdf]
